# Development and Validation of Multi-Stage Prediction Models for Pre-eclampsia: A Retrospective Cohort Study on Chinese Women

**DOI:** 10.3389/fpubh.2022.911975

**Published:** 2022-05-30

**Authors:** Zeyu Tang, Yuelong Ji, Shuang Zhou, Tao Su, Zhichao Yuan, Na Han, Jinzhu Jia, Haijun Wang

**Affiliations:** ^1^Department of Biostatistics, School of Public Health, Peking University, Beijing, China; ^2^Department of Maternal and Child Health, School of Public Health, Peking University, Beijing, China; ^3^Maternal and Child Health Care Hospital of Tongzhou District, Beijing, China; ^4^Center for Statistical Science, Peking University, Beijing, China; ^5^National Health Commission Key Laboratory of Reproductive Health, Beijing, China

**Keywords:** pre-eclampsia, multi-stage prediction model, screening strategy, pregnancy, logistic regression, LASSO

## Abstract

**Objective:**

The aim of this study is to develop multistage prediction models for pre-eclampsia (PE) covering almost the entire pregnancy period based on routine antenatal measurements and to propose a risk screening strategy.

**Methods:**

This was a retrospective cohort study that included 20582 singleton pregnant women with the last menstruation between January 1, 2013 and December 31, 2019. Of the 20582 women, 717 (3.48%) developed pre-eclampsia, including 46 (0.22%) with early-onset pre-eclampsia and 119 (0.58%) preterm pre-eclampsia. We randomly divided the dataset into the training set (*N* = 15665), the testing set (*N* = 3917), and the validation set (*N* = 1000). Least Absolute Shrinkage And Selection Operator (LASSO) was used to do variable selection from demographic characteristics, blood pressure, blood routine examination and biochemical tests. Logistic regression was used to develop prediction models at eight periods: 5–10 weeks, 11–13 weeks, 14–18 weeks, 19–23 weeks, 24–27 weeks, 28–31 weeks, 32–35 weeks, and 36–39 weeks of gestation. We calculated the AUROC (Area Under the Receiver Operating Characteristic Curve) on the test set and validated the screening strategy on the validation set.

**Results:**

We found that uric acid tested from 5–10 weeks of gestation, platelets tested at 18–23 and 24–31 weeks of gestation, and alkaline phosphatase tested at 28–31, 32–35 and 36–39 weeks of gestation can further improve the prediction performance of models. The AUROC of the optimal prediction models on the test set gradually increased from 0.71 at 5–10 weeks to 0.80 at 24–27 weeks, and then gradually increased to 0.95 at 36–39 weeks of gestation. At sensitivity level of 0.98, our screening strategy can identify about 94.8% of women who will develop pre-eclampsia and reduce about 40% of the healthy women to be screened by 28–31 weeks of pregnancy.

**Conclusion:**

We developed multistage prediction models and a risk screening strategy, biomarkers of which were part of routine test items and did not need extra costs. The prediction window has been advanced to 5–10 weeks, which has allowed time for aspirin intervention and other means for PE high-risk groups.

## Introduction

Pre-eclampsia is a pregnancy related syndrome defined as newly occurred hypertension at or after 20 weeks of gestation, accompanied by proteinuria or other organs damage (1). The incidence of pre-eclampsia worldwide is 0.2-9.2% (2). A study including 112,386 pregnant women in China showed that the incidence of pre-eclampsia in China was approximately 2.87% in 2011 (3). Every year, approximately 76 thousands women and half million infants died from pre-eclampsia worldwide. Pre-eclampsia can have adverse effects on pregnant women, for instance, causing damage to the liver and kidney systems (4). If left untreated, it can lead to pulmonary edema, eclampsia, brain damage, and even maternal death (5–7). Pregnant women and their children affected by pre-eclampsia are at increased risk for long-term cardiovascular and chronic diseases, including chronic hypertension, stroke, metabolic syndrome, and cognitive impairment (8–16).

In the first trimester, taking pharmacologic interventions (e.g., aspirin) for high-risk pregnant women can reduce the risk of early-onset and preterm pre-eclampsia (17, 18). It can reduce the incidence of adverse perinatal outcomes by intensive monitoring and selecting the appropriate time of delivery during the second or third trimester (19). Early identification of high-risk groups will help to take interventions in advance. Therefore, it is of great significance to develop risk prediction models for pre-eclampsia.

However, pre-eclampsia related prediction models have been developed mainly in developed countries (20). In recent years, there were some studies developing prediction models of pre-eclampsia based on Chinese population (21–26). These studies were mainly based on the hospital electronic medical data system and carried out in eastern China (three in Shanghai and one in Tianjin), screening pregnant women in the first and second trimester of pregnancy. However, these studies primarily focused on specific high-risk groups or used expensive biomarkers beyond the scope of routine testing, which limits their generalizability. In addition, these studies did not consider pre-eclampsia subtypes (23–26), possible bias caused by the process of variable selection (23, 24, 26), and insufficient number of outcome events (21–23, 25).

In this study, based on 20582 pregnant women in Beijing, we aimed to develop multistage prediction models covering almost the entire pregnancy period by selecting valuable predictors from routine antenatal measurements, and a risk screening strategy based on the optimal models.

## Methods

### Study Population

**Figure 1:**
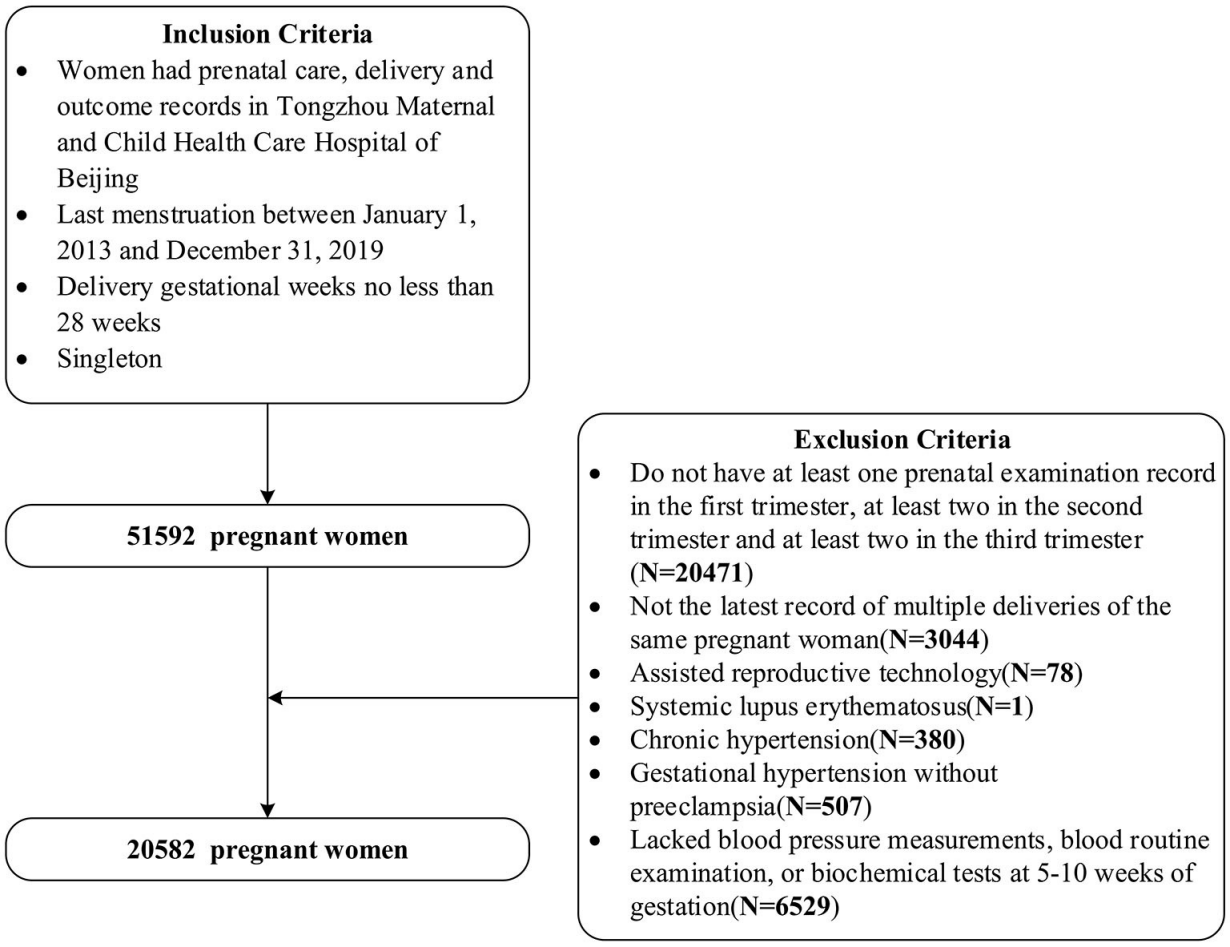
The inclusion and exclusion criteria for the study population.

This was a Peking University Retrospective Birth Cohort in Tongzhou based on the hospital information system, including singleton pregnant women having prenatal care, delivery and outcome records, with last menstruation between 1 January 2013 and 31 December 2019 and with delivery gestational weeks no less than 28 weeks in Tongzhou Maternal and Child Health Care Hospital of Beijing. We further selected pregnant women with the latest record of deliveries, and with at least one, two and two antenatal examination records in the first, second, and third trimester, respectively. We excluded pregnant women using assisted reproductive technology, having systemic lupus erythematosus, or chronic hypertension, or gestational hypertension without pre-eclampsia. Also, we excluded pregnant women who lacked blood pressure measurements, blood routine examination and biochemical tests records at 5–10 weeks of gestation. Finally, we included 20,582 women in our study. The inclusion and exclusion criteria can be seen in Figure 1. The studies involving human participants were reviewed and approved by Institutional Review Board of Peking University Health Science Center (No. IRB00001052-21023).

### Maternal Characteristics, Medical History and Biomarkers

We extracted maternal characteristics, medical history, blood pressure measurements, blood routine examination and biochemical tests from the electronic data system of Tongzhou Maternal and Child Health Hospital of Beijing. Maternal characteristics included maternal height (cm), pre-pregnancy weight (kg), pre-pregnancy BMI, pre-gestational diabetes mellitus, ethnicity, parity, gravidity, abortion history, family history of hypertension, family history of diabetes, maternal age, husband age. Blood pressure measurement records included systolic blood pressure (SBP) and diastolic blood pressure (DBP) measurement records and we calculated mean arterial pressure (MAP) by MAP = (SBP+2 × DBP)/3. Blood routine examination and biochemical tests included 48 biomarkers,including hemoglobin, mean red blood cell volume, platelet, red blood cell, white blood cell, alanine aminotransferase, aspartate aminotransferase, urea nitrogen, and calcium, etc. Lists of all biomarkers were shown in Supplementary Tables 1a,b.

### Maternal Outcomes

Based on the Guidelines on Diagnoses and Treatments of Hypertensive Disorders in Pregnancy by Chinese Society of Obstetrics and Gynecology, pre-eclampsia is defined as hypertension first appeared after 20 weeks of pregnancy, systolic blood pressure ≥140 and/or diastolic blood pressure ≥90 mmHg, with proteinuria or with any of the following organs or systems involved: heart, lung, liver, kidney and other important organs, or abnormal changes in blood system, digestive system and nervous system, placenta and fetus involved, etc (27). According to the time of diagnosis, we divided pre-eclampsia into early-onset pre-eclampsia (<34+0 weeks of gestation), preterm pre-eclampsia (<37+0 weeks of gestation), late-onset pre-eclampsia (≥34+0 weeks of gestation) and term pre-eclampsia (≥37+0 weeks of gestation).

### Statistics

#### Division of Gestational Weeks

The first trimester, the second trimester and the third trimester were defined as < = 13 weeks, 14–27 weeks and > = 28 weeks, respectively (24, 28). We divided the whole pregnancy into eight periods: 5–10 weeks, 11–13 weeks, 14–18 weeks, 19–23 weeks, 24–27 weeks, 28–31 weeks, 32–35 weeks and 36–39 weeks. Therefore, the first trimester, the second trimester and the third trimester of pregnancy included 2, 3 and 3 time periods, respectively.

#### Preprocessing Variables and Imputing Missing Values

The pregnant women's height, pre pregnancy weight, pre pregnancy BMI, pregnant women's age and husband's age were used as continuous variables. We divided pre-gestational diabetes mellitus, family history of hypertension, abortion history, family history of diabetes into yes or no and we divided ethnicity into Han nationality or other nationalities. Parity was divided into primiparous or multiparous, and gravidity was divided into the first pregnancy, the second pregnancy, the third and more pregnancies. We divided the pregnancy season into spring, summer, autumn and winter according to the date of the last menstruation after verification.

We also kept blood pressure, routine blood and biochemical tests as continuous variables. If pregnant women had more than one measurement record during a certain period, we took the average value of these records. Therefore, regardless of missing values, each pregnant woman should have eight records of mean arterial pressure and biomarker measurements throughout pregnancy. There was one measurement record in each of the eight time periods. In particular, pregnant women rarely did biochemical tests in the second trimester of pregnancy. Therefore, there was no such test value in the 14–18 weeks, 19–23 weeks, and 24–27 weeks, so the variable was marked as missing.

The missing rate of demographic characteristic variables was <2%. We used the median to impute the missing values of continuous variables and the mode to impute the missing values of categorical variables. For the mean arterial pressure measurements, blood routine examination and biochemical tests (except the second trimester of pregnancy), the missing values were imputed by the values in the previous period. For example, missing values at 32–35 weeks of gestation were imputed by the measurements at 28–31 weeks of gestation. A pregnant woman has a prenatal examination at 32–35 weeks of pregnancy, but for some reason, she lacked a blood routine examination. Then, this missing value can be replaced by the value tested during 28–31 weeks. In clinical application, this method is easy for health professionals to understand and use it. If a pregnant woman is unable to get biochemical indicators for some reasons, this method can be used to quickly impute the missing value. In addition, by using previous measurements, this method can avoid reverse causality. The missing data imputed by the last observation carried forward (LOCF) method was used in many clinical antidepressant trials and other high level clinical studies (29–31).

#### Development and Validation of Prediction Model

We sampled from women with and without pre-eclampsia to form a training set (*N* = 15665), a test set (*N* = 3917) and a validation set (*N* = 1000). The incidence rate of pre-eclampsia was almost the same in each data set (3.5%). We developed prediction models on the training set and verify the model on the test set. Based on the calculation results from the training set and test set, a screening strategy was formed and tested on the validation set. We did univariate analysis on demographic characteristics to compare the differences between each group and the control group (women without any types of pre-eclampsia), for example, comparison of women with early-onset pre-eclampsia and without any types of pre-eclampsia. We used Chi-test for categorical variables (Fisher exact test when the counts in some cells was fewer than five) and Kruskal-Wallis Rank Sum test for continuous variables.

We developed prediction models by using Logistic Regression on the imputed training set and calculated the area under receiver operator curve (AUROC) on the test set to reflect the ability of prediction. We used LASSO technique to select the variables with the highest priority and the second priority to join in the model according to the order in which the coefficients enter into the model along the solution path.

Least Absolute Shrinkage And Selection Operator (LASSO) is a shrinking technique used to do variable selection. It has been used to do variable selection and avoid overfitting for development of pre-eclampsia prediction models in previous studies (25, 32). LASSO was described as follows. For more details, we recommended readers to related materials (33, 34).

The objective of LASSO is to find the proper coefficients to minimize the loss function:


(1) $$\begin{array}{r} \text{min}\;-\;\frac{1}{N}\Sigma_{i\;=\;1}^{N}l\left(y_{i}|{\text{X}}_{i}\right)+\lambda \Sigma_{j\;=\;1}^{J}|\beta_{j}|, \end{array}$$

where **X**_*i*_ = (*x*_*i*1_, *x*_*i*2_, ……, *x*_*iN*_) is the vector of observed variables of the subject *i*, *y*_*i*_ is the outcome of the subject *i*, *y*_*i*_ϵ{0, 1}, *y*_*i*_ = 1 if the subject *i* developed pre-eclampsia and *y*_*i*_ = 0 if the subject *i* did not develop pre-eclampsia. |β_*j*_| is the absolute value of the coefficient of the variable *x*_*ij*_. λ is a tuning parameter: when it is big enough, all coefficients are zeros and the coefficients gradually turn to non-zero with the decrease of λ. *l*(*y*_*i*_|**X**_*i*_) is the log-likelihood function of the logistic regression, given by the following equation:


(2) $$\begin{array}{l} l\left(y_{i}|X_{i}\right)\;=\;y_{i}\cdot \;\left(\beta_{0}\;+\;\Sigma_{j\;=\;1}^{J}\beta_{j}x_{ij}\right)\\ \;-\;\log\left[1\;+\;\exp\left(\beta_{0}\;+\;\Sigma_{j\;=\;1}^{J}\beta_{j}x_{ij}\right)\right]. \end{array}$$

We determined a basic variable set, which was forced into models, and then selected the variables that can improve AUROC on the test set from the demographical variables, mean arterial pressure and biochemical markers in proper order in four stages. We developed predictions on each period (5–10 weeks, 11–13 weeks, 14–18 weeks, 19–23 weeks, 24–27 weeks, 28–31 weeks, 32–35 weeks and 36–39 weeks). The four stages of developing models for each period were described as follows.

#### Stage 1

As reported in previous studies, pre-pregnancy BMI, pre-gestational diabetes mellitus (PGDM), parity, family history of hypertension and maternal age were highly associated with pre-eclampsia, thus these five variables were forced into models and denoted as “basic variables” (1, 35, 36). Then, we added other demographical variables and medical history into the model, including maternal height, pre-pregnancy weight (kg), ethnicity, gravidity, abortion history, husband age (years), and pregnancy season. Last, we selected the optimal set of variables with the highest AUROC denoted as Stage1-Optimal Variables.

#### Stage 2

Based on Stage1-Optimal Variables, we added MAPs measured at baseline (5–10 weeks) and the current period and then tested whether MAPs could improve the ability of prediction. For example, for women took prenatal examinations during 28–31 weeks, we added MAPs measured at 5–10 weeks and 28–31 weeks into the model. The optimal set of variables with the highest AUROC was denoted as Stage2-Optimal Variables.

#### Stage 3

Based on Stage2-Optimal Variables, we added biomarkers measured at baseline (5–10 weeks) and the current period. By using LASSO, we selected the optimal set of variables with the highest AUROC denoted as Stage3-Optimal Variables. As mentioned before, we did not add biochemical tests measured in the second trimester, because it was rarely tested in the hospital.

#### Stage 4

At the current period, we further added all MAPs measured in previous periods based on Stage2-Optimal Variables and added all biomarkers measured in previous periods based on Stage3-Optimal Variables to check whether adding other variables could improve the AUROC.

We excluded women who had used aspirin during pregnancy and conducted sensitivity analysis by redeveloping optimal prediction models to test whether the valuable biomarkers we found could still improve the predictive ability of models (37).

After developing the best prediction model at each stage, we selected the appropriate risk cutoff values to make the sensitivity of the prediction models reach 0.95, 0.96, 0.97, 0.98, and 0.99 in each period. Then, we screened the population in the validation set with different sensitivities to form the best screening strategy.

We used R software (38) to do all calculations. The “glmnet” package in R was used to fit logistic regressions via LASSO (39). Details about how to perform it in R are available at vignettes of the “glmnet” package.

## Results

### Comparison of Characteristics Between Women With and Without Pre-eclampsia

Table 1 showed demographical characteristics of women with and without pre-eclampsia. Compared with those without pre-eclampsia, women with pre-eclampsia had higher body weight and body mass index (BMI), and had higher proportion of pre-gestational diabetes, primipara, first pregnancy, family history of hypertension, family history of diabetes, pregnancy in spring and winter, and gestational diabetes. The differences between the two groups were statistically significant. Similar results were seen in women with early-onset and preterm pre-eclampsia. However, there were not obvious difference in the proportion of pre-gestational diabetes mellitus, family history of hypertension, family history of diabetes, and pregnancy season between women with and without early-onset pre-eclampsia, between women with and without preterm pre-eclampsia.

**Table 1:** Characteristics of pregnancies in imputed datasets.

	**Without pre-eclampsia**	**All pre-eclampsia**	***P*** **-value**	**Early-onset** **pre-eclampsia**	***P*** **-value**	**Preterm** **pre-eclampsia**	***P*** **-value**	**Late-onset pre-eclampsia**	***P*** **-value**	**Term pre-eclampsia**	***P*** **-value**
Nunber	19865	717		46		119		671		598	
Maternal Height (median [IQR])	162.00 [160.00, 165.00]	161.00 [158.00, 165.00]	0.19	161.00 [160.00, 164.75]	0.797	160.00 [158.00, 165.00]	0.105	161.00 [158.00, 165.00]	0.197	161.00 [159.00, 165.00]	0.469
Pre-pregnancy Weight (median [IQR])	57.00 [52.00, 63.00]	60.00 [54.50, 70.00]	<0.001	63.70 [56.00, 71.50]	<0.001	62.00 [55.00, 70.00]	<0.001	60.00 [54.00, 70.00]	<0.001	60.00 [54.00, 70.00]	<0.001
Pre-pregnancy BMI (median [IQR])	21.63 [19.81, 23.88]	23.44 [20.96, 26.53]	<0.001	23.97 [21.67, 27.50]	<0.001	24.03 [21.54, 26.72]	<0.001	23.44 [20.95, 26.39]	<0.001	23.44 [20.89, 26.36]	<0.001
Pre-gestational diabetes mellitus (%)			<0.001		0.274		0.563				<0.001
No	19728 (99.3)	702 (97.9)		45 (97.8)		118 (99.2)		657 (97.9)	<0.001	584 (97.7)	
Yes		15 (2.1)		1 (2.2)		1 ( 0.8)		14 (2.1)		14 (2.3)	
Ethnicity (%)			1		0.757		1		1		1
Han	18663 (93.9)	674 (94.0)		43 (93.5)		112 (94.1)		631 (94.0)		562 (94.0)	
Others	1202 (6.1)	43 (6.0)		3 (6.5)		7 (5.9)		40 (6.0)		36 (6.0)	
Parity (%)			<0.001		0.012		0.018		<0.001		<0.001
Primiparous	12165 (61.2)	534 (74.5)		37 (80.4)		86 (72.3)		497 (74.1)		448 (74.9)	
Multiparous	7700 (38.8)	183 (25.5)		9 (19.6)		33 (27.7)		174 (25.9)		150 (25.1)	
Gravidity (%)			<0.001		0.011		0.49		<0.001		<0.001
1	8455 (42.6)	366 (51.0)		29 (63.0)		57 (47.9)		337 (50.2)		309 (51.7)	
2	6345 (31.9)	187 (26.1)		12 (26.1)		36 (30.3)		175 (26.1)		151 (25.3)	
≧3	5065 (25.5)	164 (22.9)		5 (10.9)		26 (21.8)		159 (23.7)		138 (23.1)	
Abortion History (%)			0.898		0.051		0.707		0.742		1
No	12101 (60.9)	439 (61.2)		35 (76.1)		75 (63.0)		404 (60.2)		364 (60.9)	
Yes	7764 (39.1)	278 (38.8)		11 (23.9)		44 (37.0)		267 (39.8)		234 (39.1)	
Family history of hypertension (%)			0.034		1		0.636		0.017		0.008
No	19064 (96.0)	676 (94.3)		45 (97.8)		116 (97.5)		631 (94.0)		560 (93.6)	
Yes	801 (4.0)	41 (5.7)		1 (2.2)		3 (2.5)		40 (6.0)		38 (6.4)	
Family history of diabetes (%)			0.033		0.566		0.477		0.038		0.042
No	19508 (98.2)	696 (97.1)		45 (97.8)		116 (97.5)		651 (97.0)		580 (97.0)	
Yes	357 (1.8)	21 (2.9)		1 (2.2)		3 (2.5)		20 (3.0)		18 (3.0)	
Maternal age (median [IQR])	29.00 [27.00, 32.00]	29.00 [26.00, 32.00]	0.763	28.00 [26.25, 31.00]	0.652	29.00 [27.00, 32.00]	0.416	29.00 [26.50, 32.00]	0.845	29.00 [26.00, 32.00]	0.49
Husband age (median [IQR])	30.00 [27.00, 33.00]	30.00 [27.00, 33.00]	0.323	30.00 [27.25, 33.00]	0.995	30.00 [28.00, 33.00]	0.617	30.00 [27.00, 33.00]	0.306	30.00 [27.00, 33.00]	0.192
Pregnancy season (%)			0.001		0.328		0.401		0.002		0.003
Spring	5006 (25.2)	218 (30.4)		15 (32.6)		36 (30.3)		203 (30.3)		182 (30.4)	
Summer	4746 (23.9)	162 (22.6)		13 (28.3)		27 (22.7)		149 (22.2)		135 (22.6)	
Autumn	4601 (23.2)	128 (17.9)		6 (13.0)		21 (17.6)		122 (18.2)		107 (17.9)	
Winter	5512 (27.7)	209 (29.1)		12 (26.1)		35 (29.4)		197 (29.4)		174 (29.1)	
Gestational diabetes mellitus			<0.001		0.025		0.001		<0.001		<0.001
	14749 (74.2)	460 (64.2)		27 (58.7)		72 (60.5)		433 (64.5)		388 (64.9)	
	5116 (25.8)	257 (35.8)		19 (41.3)		47 (39.5)		238 (35.5)		210 (35.1)	

*P-values were calculated when the reference group is the one for women without any pre-eclampsia*.

### Results of Variables Selection

#### Results of Stage 1

Supplementary Tables 2a,b, showed that before 20 weeks of gestation, the AUROC of the model on basic variables was 0.68 for all pre-eclampsia, 0.73 and 0.74 for early-onset and preterm pre-eclampsia, respectively. The AUROC of late-onset pre-eclampsia and term preeclampsia were 0.68 and 0.67, respectively. The model developed during 24–27 weeks had similar results. However, additional demographical characteristics before 20 weeks of gestation and gestational diabetes mellitus at 24–27 weeks of gestation did not increase AUROC on the test set. The Stage1-Optimal Variables included pre-pregnancy BMI, pre-gestational diabetes mellitus (PGDM), parity, family history of hypertension and maternal age.

#### Results of Stage 2

Tables 2A,B showed the results of Stage 2. For all pre-eclampsia, the AUROC increased from 0.68 to 0.70 with the addition of mean arterial pressure (MAP) measured at 5–10 weeks. With the increase of gestational weeks, the AUROC gradually increased to 0.78 with addition of MAPs measured at 5–10 and 28–31 weeks, and remained stable at 32–35 weeks and 36–39 weeks. Similar trends were seen in the late-onset and term pre-eclampsia, where the AUROC gradually increased from around 0.68 to 0.77.

**Table 2A:** Performance of prediction model based on characteristics, medical history, mean arterial pressure, and biomarkers for all, early-onset and preterm pre-eclampsia.

**Gestational age of prediction (weeks)**	**Variables**	**All pre-eclampsia**				**Early-onset pre-eclampsia**				**Preterm pre-eclampsia**			
		**Sample size**	**Cases (*N*)**	**Cases (%)**	**AUROC**	**Sample size**	**Cases (*N*)**	**Cases (%)**	**AUROC**	**Sample size**	**Cases (*N*)**	**Cases (%)**	**AUROC**
<20	Basic Variables	15665	548	3.50%	0.70	15665	39	0.25%	0.82	15665	95	0.61%	0.78
5–10	+MAP (5–10)	15665	548	3.50%	0.70	15665	39	0.25%	0.82	15665	95	0.61%	0.78
	+MAP (5–10)+uric acid (5–10)	15665	548	3.50%	0.71	15665	39	0.25%	0.82	15665	95	0.61%	0.80
11–13	+MAPs (5–10,11–13)	15665	548	3.50%	0.74	15665	39	0.25%	0.78	15665	95	0.61%	0.77
	+MAPs (5–10,11–13)+uric acid (11–13)	15665	548	3.50%	0.75	15665	39	0.25%	0.78	15665	95	0.61%	0.78
14–18	+MAPs (5–10,14–18)	15665	548	3.50%	0.76	15665	39	0.25%	0.87	15665	95	0.61%	0.81
19–23	+MAPs (5–10,19–23)	15655	538	3.44%	0.76	15655	32	0.20%	0.83	15655	88	0.56%	0.81
	+MAPs (5–10,19–23)+platelets(5–10)+platelets (19–23)	15655	538	3.44%	0.79	15655	32	0.20%	0.85	15655	88	0.56%	0.83
24–27	+MAPs (5–10,24–27)	15644	527	3.37%	0.77	15644	25	0.16%	0.85	15644	81	0.52%	0.82
	+MAPs (5–10,24–27)+platelets (5–10)+platelets (24–27)	15644	527	3.37%	0.80	15644	25	0.16%	0.86	15644	81	0.52%	0.84
28–31	+MAPs (5–10,28–31)	15619	504	3.23%	0.78	15619	10	0.06%	0.89	15619	65	0.42%	0.80
	+MAPs (5–10,28–31)+uric acid (28–31)	15619	504	3.23%	0.79	15619	10	0.06%	0.89	15619	65	0.42%	0.82
	+MAPs (5–10,28–31)+alkaline phosphatase (28–31)	15619	504	3.23%	0.85	15619	10	0.06%	0.94	15619	65	0.42%	0.83
	+MAPs (5–10,28–31)+uric acid(28–31)+alkaline phosphatase (28–31)	15619	504	3.23%	0.86	15619	10	0.06%	0.96	15619	65	0.42%	0.84
32–35	+MAPs (5–10,32–35)	15478	459	2.97%	0.77								
	+MAPs (5–10,32–35)+uric acid (32–35)	15478	459	2.97%	0.80								
	+MAPs (5–10,32–35)+alkaline phosphatase (32–35)	15478	459	2.97%	0.87								
	+MAPs (5–10,32–35)+uric acid (32–35)+alkaline phosphatase (32–35)	15478	459	2.97%	0.89								
36–39	+MAPs (5–10,36–39)	10912	131	1.20%	0.78								
	+MAPs (5–10,36–39)+uric acid (36–39)	10912	131	1.20%	0.82								
	+MAPs (5–10,36–39)+alkaline phosphatase (36–39)	10912	131	1.20%	0.93								
	+MAPs (5–10,36–39)+alkaline phosphatase (36–39)+uric acid (36–39)	10912	131	1.20%	0.95								

*Basic Variables include pre-pregnancy BMI, pre-gestational diabetes mellitus, parity, family history of hypertension and maternal age, which are forced into models. MAP, Mean Arterial Pressure; Numbers in brackets represent the gestational age when MAPS and biomarkers were tested*.

**Table 2B:** Performance of prediction model based on characteristics, medical history, mean arterial pressure, and biomarkers for late-onset and term pre-eclampsia.

**Gestational age of predictio*n* (weeks)**	**Variables**	**Late-onset pre-eclampsia**				**Term pre-eclampsia**			
		**Sample Size**	**Cases (*N*)**	**Cases (%)**	**AUROC**	**Sample size**	**Cases (*N*)**	**Cases (%)**	**AUROC**
<20	Basic variables	15665	509	3.25%	0.69	15665	453	2.89%	0.68
5–10	+MAP (5–10)	15665	509	3.25%	0.69	15665	453	2.89%	0.68
	+MAP (5–10)+uric acid (5–10)	15665	509	3.25%	0.71	15665	453	2.89%	0.69
11–13	+MAPs (5–10,11–13)	15665	509	3.25%	0.73	15665	453	2.89%	0.73
	+MAPs (5–10, 11–13)+uric acid (11–13)	15665	509	3.25%	0.75	15665	453	2.89%	0.74
14–18	+MAPs (5–10,14–18)	15665	509	3.25%	0.75	15665	453	2.89%	0.75
19–23	+MAPs (5–10,19–23)	15655	506	3.23%	0.76	15655	450	2.87%	0.75
	+MAPs (5–10,19–23)+platelets (5–10)+platelets (19–23)	15655	506	3.23%	0.79	15655	450	2.87%	0.78
24–27	+MAPs (5–10,24–27)	15644	502	3.21%	0.76	15644	446	2.85%	0.76
	+MAPs (5–10,24–27)+platelets (5–10)+platelets (24–27)	15644	502	3.21%	0.80	15644	446	2.85%	0.79
28–31	+MAPs (5–10,28–31)	15619	494	3.16%	0.77	15619	439	2.81%	0.77
	+MAPs (5–10,28–31)+uric acid (28–31)	15619	494	3.16%	0.79	15619	439	2.81%	0.78
	+MAPs (5–10,28–31)+alkaline phosphatase (28–31)	15619	494	3.16%	0.84	15619	439	2.81%	0.84
	+MAPs (5–10,28–31)+uric acid (28–31)+alkaline phosphatase (28–31)	15619	494	3.16%	0.86	15619	439	2.81%	0.86
32–35	+MAPs (5–10,32–35)					15478	429	2.77%	0.77
	+MAPs (5–10,32–35)+uric acid (32–35)					15478	429	2.77%	0.80
	+MAPs (5–10,32–35)+alkaline phosphatase (32–35)					15478	429	2.77%	0.87
	+MAPs (5–10,32–35)+uric acid (32–35)+alkaline phosphatase (32–35)					15478	429	2.77%	0.89
36–39	+MAPs (5–10,36–39)								
	+MAPs (5–10,36–39)+uric acid (36–39)								
	+MAPs (5–10,36–39)+alkaline phosphatase (36–39)								
	+MAPs (5–10,36–39)+alkaline phosphatase (36–39)+uric acid (36–39)								

*Basic Variables include pre–pregnancy BMI, pre–gestational diabetes mellitus, parity, family history of hypertension and maternal age, which are forced into models. MAP, Mean Arterial Pressure. Numbers in brackets represent the gestational age when MAPS and biomarkers were tested*.

For early-onset and preterm pre-eclampsia, AUROC gradually increased from about 0.73 to 0.89 and 0.80 at 28–31 weeks of gestation, respectively. Generally, based on the Stage 1-Optimal Variables, the addition of MAPs increased AUROC for all pre-eclampsia and its subtypes, and the AUROC was higher for early-onset and preterm preeclampsia than that for late-onset and term pre-eclampsia. The Stage 2-Optimal Variables included Stage 1-Optimal Variables and MAPs measured at baseline (5–10 weeks) and at the current prenatal examination period.

#### Results of Stage 3

As can be seen in Tables 2A,B, based on Stage 2-Optimal Variables, we found that the AUROC of all types of pre-eclampsia can be slightly improved by adding uric acid test in 5–10 weeks and 11–13 weeks of gestation. At 18–23 weeks and 24–27 weeks of gestation, the addition of platelets tested at baseline and the current period can improve AUROC for all types of pre-eclampsia (0.79–0.86). Also, we found that adding uric acid and alkaline phosphatase tested in the current period at 28-31 weeks, 32–35 weeks and 36–39 weeks of pregnancy can significantly improve the prediction ability for all types of pre-eclampsia, especially for pre-eclampsia occurred after 31 weeks (including late-onset and term pre-eclampsia). For all pre-eclampsia, the AUROC increased from approximately 0.78 to 0.86 (28–31 weeks), 0.89 (32–35 weeks) and 0.95 (36–39 weeks), respectively. Stage3-Optimal Variables are as follows: (i) Stage2-Optimal Variables plus uric acid tested in the current period in 5–10 weeks and 11–13 weeks; (ii) Stage 2-Optimal Variables without adding biomarkers in 14–18 weeks; (iii) Stage 2-Optimal Variables plus platelets tested at baseline and the current period in 18–23 weeks and 24–27 weeks; (iv) Stage 2-Optimal Variables plus uric acid and alkaline phosphatase tested in the current period at 28–31 weeks, 32–35 weeks and 36–39 weeks.

#### Results of Stage 4

As can be seen in Supplementary Tables 3a,b, we found that adding the MAPs measured in all previous periods to Stage 2-Optimal Variables and adding biomarkers tested in all previous periods to Stage 3-Optimal Variables almost did not improve AUROC. In order to prevent over fitting, we chose Stage 3-Optimal Variables to develop our final predictions models.

#### Results of Sensitivity Analysis

There were 104 and 28 women using aspirin in the training set (N = 15665) and test set (*N* = 3917), respectively. For the optimal prediction models in each stage, the results were very similar in the datasets with and without women who had used aspirin, which were shown in Supplementary Tables 4a–e. Although a few women had used aspirin during pregnancy, it was less likely to affect our results.

### Best Prediction Model in Each Period

#### The Best Prediction Models in Each Period Are Shown as Below

The risk score for pre-eclampsia of each woman is calculated by odds/(1+odds), where odds = e^Y^.

#### Period 1 (5–10 Weeks)

Y = (−13.40) + (0.102^*^BMI) + (−0.920 if Multiparous) + (0.0445^*^ Maternal Age) + (0.433^*^ if Pre–gestational diabetes mellitus) + (0.320^*^ if Family history of hypertension) + (0.0551^*^).

#### Period 2 (11–13 Weeks)

Y = (−15.10) + (0.0890^*^BMI) + (−0.888 if Multiparous) + (0.0479^*^ Maternal Age) + (0.407^*^ if Pre–gestational diabetes mellitus) + (0.319^*^ if Family history of hypertension) + (0.0233^*^ MAP measured at 5–10 weeks) + (0.0503^*^ MAP measured at 11–13 weeks) + (0.00609^*^ uric acid test at 11–13 weeks).

#### Period 3 (14–18 Weeks)

Y = (−15.70) + (0.110^*^BMI) + (−0.903 if Multiparous) + (0.0460^*^ Maternal Age) + (0.499^*^ if Pre-gestational diabetes mellitus) + (0.347^*^ if Family history of hypertension) + (0.0182^*^ MAP measured at 5–10 weeks) + (0.0704^*^ MAP measured at 14–18 weeks).

#### Period 4 (19–23 Weeks)

Y = (−15.80) + (0.110^*^BMI) + (−0.917 if Multiparous) + (0.0531^*^ Maternal Age) + (0.453^*^ if Pre-gestational diabetes mellitus) + (0.411^*^ if Family history of hypertension) + (0.0253^*^ MAP measured at 5–10 weeks) + (0.0648^*^ MAP measured at 19–23 weeks) + (−0.0237^*^ platelets test at 5–10 weeks) + (0.0231^*^ platelets test at 19–23 weeks).

#### Period 5 (24–27 Weeks)

Y = (−16.10) + (0.110^*^BMI) + (−0.929 if Multiparous) + (0.0531^*^ Maternal Age) + (0.400^*^ if Pre–gestational diabetes mellitus) + (0.408^*^ if Family history of hypertension) + (0.0274^*^ MAP measured at 5–10 weeks) + (0.0664^*^ MAP measured at 24–27 weeks) + (−0.0222^*^ platelets test at 5–10 weeks) + (0.0217^*^ platelets test at 24–27 weeks).

#### Period 6 (28–31 Weeks)

Y = (−15.60) + (0.0778^*^BMI) + (−0.941 if Multiparous) + (0.0611^*^ Maternal Age) + (0.410^*^ if Pre-gestational diabetes mellitus) + (0.375^*^ if Family history of hypertension) + (0.0262^*^ MAP measured at 5–10 weeks) + (0.0778^*^ MAP measured at 28–31 weeks) + (0.00974^*^ uric acid test at 28–31 weeks) + (−0.0578^*^ alkaline phosphatase test at 28–31 weeks).

#### Period 7 (32–35 Weeks)

Y = (−16.00) + (0.0586^*^BMI) + (−0.968 if Multiparous) + (0.0445^*^ Maternal Age) + (0.509^*^ if Pre–gestational diabetes mellitus) + (0.455^*^ if Family history of hypertension) + (0.0203^*^ MAP measured at 5–10 weeks) + (0.110^*^ MAP measured at 32–35 weeks) + (0.00938^*^ uric acid test at 32–35 weeks) + (−0.0751^*^ alkaline phosphatase test at 32–35 weeks).

#### Period 8 (36–39 Weeks)

Y = (−14.90) + (0.0252^*^BMI) + (−1.430 if Multiparous) + (0.0357^*^ Maternal Age) + (0.617^*^ if Pre-gestational diabetes mellitus) + (0.332^*^ if Family history of hypertension) + (−0.0144^*^ MAP measured at 5–10 weeks) + (0.129^*^ MAP measured at 36–39 weeks) + (0.0122^*^ uric acid test at 36–39 weeks) + (−0.0600^*^ alkaline phosphatase test at 36–39 weeks).

### Screening Strategy

**Figure 2:**
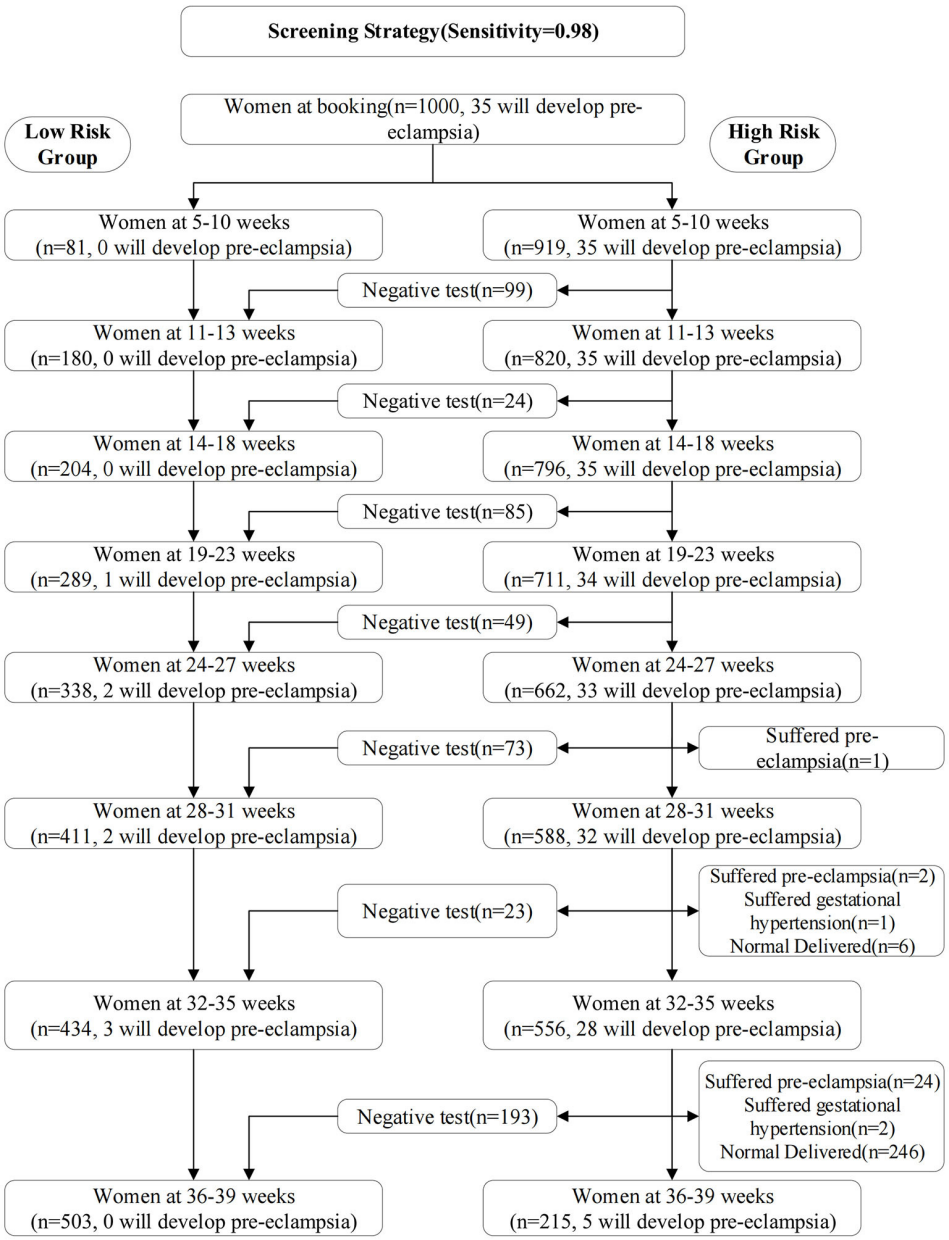
Screening results of the validation set using the optimal prediction models with the sensitivity of 0.98. From 5–10 weeks of gestation, 1000 women participated in prenatal examination.

The screening strategy was shown in Table 3 and Figure 2. We selected the 0.01, 0.011, 0.008, 0.009, 0.01, 0.003, 0.001, and 0.002 as risk cutoff values for each stage to make the sensitivity reach 0.98 (Table 3). Figure 2 showed the screening results of the validation set using the optimal prediction models with the sensitivity of 0.98. From 5–10 weeks of gestation, 1000 women participated in prenatal examination. Risk scores were calculated by the optimal prediction models for each pregnant woman participating in prenatal examination. Pregnant women exceeding the risk cutoff value were classified as high-risk group, and the risk assessment will be carried out in the next prenatal examination period; Otherwise, they were classified as the low-risk group and will not be examined in subsequent prenatal examinations. According to this method, after the prenatal examination of 14–18 weeks of pregnancy, 796 women were divided into the high-risk group, of which 35 will develop pre-eclampsia, and 204 women were divided into the low-risk group, of which 0 will develop pre-eclampsia. The number of women who need to continue screening decreased by about 20%. After the prenatal examination at 32–35 weeks of gestation, 556 women were divided into high-risk group, of which 28 will develop pre-eclampsia; 434 women were divided into the low-risk group, of which 3 will develop pre-eclampsia. The number of women who need continue screening decreased by about 45%.

**Table 3:** The optimal multi–stage prediction models with the sensitivity of 0.98.

**Gestational age of prediction (weeks)**	**Variables**	**Sample size**	**Cases (*N*)**	**Cases (%)**	**AUROC**	**Risk score threshold**	**Senstivity**	**Specifity**	**Positive predictive value**	**Negative predictive value**
5–10	Basic variables+MAP (5–10)+uric acid (5–10)	15665	548	3.50%	0.71	0.010	0.985	0.091	0.037	0.994
11–13	Basic Variables+MAPs (5–10,11–13)+uric acid (11–13)	15665	548	3.50%	0.75	0.011	0.978	0.164	0.040	0.995
14–18	Basic Variables+MAPs (5–10,14–18)	15665	548	3.50%	0.76	0.008	0.978	0.113	0.038	0.993
19–23	Basic Variables+MAPs (5–10,19–23)+platelets (5–10)+platelets (19–23)	15655	538	3.44%	0.79	0.009	0.985	0.195	0.041	0.997
24–27	Basic Variables+MAPs (5–10,24–27)+platelets (5–10)+platelets (24–27)	15644	527	3.37%	0.80	0.010	0.985	0.239	0.042	0.998
28–31	Basic Variables+MAPs (5–10,28–31)+uric acid (28–31)+alkaline phosphatase (28–31)	15619	504	3.23%	0.86	0.003	0.977	0.180	0.039	0.996
32–35	Basic Variables+MAPs (5–10,32–35)+uric acid(32–35)+alkaline phosphatase(32–35)	15478	459	2.97%	0.89	0.001	0.991	0.176	0.035	0.998
36–39	Basic Variables+MAPs (5–10,36–39)+alkaline phosphatase (36–39)+uric acid (36–39)	10912	131	1.20%	0.95	0.002	0.974	0.644	0.038	0.999

*Basic Variables include pre-pregnancy BMI, pre-gestational diabetes mellitus, parity, family history of hypertension and maternal age, which are forced into models. MAP, Mean Arterial Pressure. Numbers in brackets represent the gestational age when MAPS and biomarkers were tested*.

## Discussion

### Summary

In this study, we established multi-stage pre-eclampsia risk prediction models throughout pregnancy based on 20582 pregnant women in China. We sequentially select valuable variables from demographical characteristics, mean arterial pressure, blood routine examination, and biochemical biomarkers to the prediction models. We found that uric acid tested from 5–10 weeks of gestation, platelets tested at 18-23 and 24–31 weeks of gestation, and alkaline phosphatase tested at 28–31, 32–35 and 36–39 weeks of gestation can further improve the prediction performance of models. The AUROC of the optimal prediction models on the test set gradually increased from 0.71 at 5–10 weeks to 0.80 at 24–27 weeks, and then gradually increased to 0.95 at 36–39 weeks of gestation. Based on the optimal prediction models, we established a multi-stage screening strategy from 5–10 weeks of pregnancy, which can add about 94.3% of women who will develop pre-eclampsia and reduce about 40% of the healthy women to be screened by 28–31 weeks of pregnancy.

### Comparison With Previous Studies

Some studies used demographic characteristics and medical history to build prediction models. The AUROC of prediction models developed by David Wright et al. for all pre-eclampsia, preterm pre-eclampsia, and early-onset pre-eclampsia were 0.76, 0.79, and 0.81, respectively, which were higher than 0.68, 0.74, and 0.73 in our study (40). Similar results were seen in models given by LCY Poon et al. (41). This may be because more high-risk groups were included in these two studies, for example, the model established by David Wright et al. included high-risk groups such as chronic hypertension, systemic lupus erythematosus or antiphospholipid syndrome, and assisted reproduction. The prediction model may perform better on high-risk groups.

We found that on the basis of demographic characteristics and medical history, for all pre-eclampsia, the addition of mean arterial pressure can improve the AUROC of the model. The later the gestational week for the measurement of MAPs, the higher the AUROC. It gradually increased from 0.68 to 0.78 at 28–31 weeks of gestation, and then remained stable at 32–35 weeks and 36–39 weeks of gestation. This finding is basically consistent with previous studies (42–44). Based on 12996 British women, Wallis et al. found that the AUROC increased from 0.79 to 0.80, 0.80, 0.84, 0.84, 0.84, 0.86, and 0.88 after adding the mean arterial pressure measured at 20, 25, 28, 31, 34 and 36 weeks to models with demographic characteristics, medical history, and MAP measured at baseline (43). Tayyar A. et al. also found the predictive value of adding MAPs at 12, 22, 32 and 36 weeks of gestation (44).

We found that uric acid tested from 5–10 weeks of gestation, platelets tested at 18–23 and 24–31 weeks of gestation, and alkaline phosphatase tested at 28–31, 32–35 and 36–39 weeks of gestation can further improve the prediction performance of models. There are some potential explanations for uric acid, platelets, and alkaline phosphatase to improve prediction performance. Platelets and uric acid have predictive values for pre-eclampsia. A systematic review of 69 studies showed that pregnant women with pre-eclampsia had a higher average platelet volume than the normal (45). Placental related diseases are associated with the transitional activation of maternal platelets, such as preeclampsia (46). Therefore, the detection of platelet function and over activation has a certain predictive value for preeclampsia (47). There was a strong association between uric acid and pre-eclampsia (48). The increased blood pressure can lead to organ damage, such as liver or kidney damage. Renal injury may increase the level of serum uric acid, and then increase the risk of pre-eclampsia (49, 50). It was reported that pregnant women with subsequent preeclampsia had elevated uric acid levels as early as 10 weeks of gestation (51). This phenomenon is basically consistent with the findings of this study. For alkaline phosphatase, some studies showed that the level of alkaline phosphatase was higher in women with pre-eclampsia than that in normal women (52, 53). Alkaline phosphatase may be related to pre-eclampsia, but it is necessary to further study its predictive value.

### Comparison With Chinese Population-Based Research

In recent years, there were some studies developing prediction models of pre-eclampsia based on Chinese population (21–26). These studies were mainly based on the hospital electronic medical record data system and mainly carried out in eastern China (three in Shanghai, one in Tianjin and one in Shanxi), screening pregnant women in the first and second trimester of pregnancy. Two studies predicted early-onset pre-eclampsia where the detection rate was between 40.7%−73.2% with the false positive rate of 10%. Four studies predicted all pre-eclampsia (undifferentiated subtypes), with AUROC from 0.86 to 0.98.

However, the populations and predictors used in these studies were quite different. Jiang et al. used demographic characteristics and biochemical markers (a complete blood count, serum albumin, serum uric acid, 24-h urinary protein, antinuclear antibodies, anti–double-stranded DNA antibody, antiphospholipid antibodies, etc.) to achieve an AUROC of 0.975 for pregnant women with systemic lupus erythematosus (23). Chen et al. predicted the risk of early-onset preeclampsia for pregnant women with twin pregnancy, where the AUROC was 0.82 (95% CI: 0.76–0.88), and the detection rate was 40.7% (false positive 10%) (22). Wang et al. developed a prediction model for pre-eclampsia using 31 blood flow related parameters such as vascularization index (VI), blood flow index (FI) and vascularization blood flow index (VFI) related to uterus and placenta, and the AUROC reached 0.877 (25). However, these studies aimed at specific high-risk groups, or used expensive indicators that were not within the scope of routine testing, so it may not be conducive to promotion. In addition, these studies also have other limitations: not consider pre-eclampsia subtypes (23–26), possible bias caused by variable screening process (23, 24, 26), and insufficient number of outcome events (21–23, 25).

#### Compared With the Screening Strategies of Previous Studies

Wallis et al. proposed a multi-stage screening strategy based on the British women: the first screening was conducted at < 18 weeks of gestation, and the second to fifth screening was conducted by using mean arterial pressure at 20, 25, 28, and 31 weeks of gestation. We and Wallis et al. tested the screening strategy based on the same number of pregnant women. Compared with the screening strategy proposed by Wallis et al., we advanced the prediction window as early as 5–10 weeks. After completing the screening at 28–31 weeks, we reduced the number of people to be screened by about 45%, while Wallis et al. reduced by about 35%. In particular, we extended the screening period to 36–39 weeks, which may effectively screen term pre-eclampsia and even later pre-eclampsia. Although the two strategies target different populations and it is necessary to compare the screening strategies of the two sides in the same population in the future, our screening strategy has the potential for improvement.

### Advantages, Clinical Value and Limitations of the Study

We have several advantages in our study. First, our sample size and the number of outcomes were larger than that in previous prediction models based on Chinese population. Second, compared with the stepwise regression used in many studies, we used LASSO for variable selection, so it is less likely to over fitting, and we tested our model on the randomly divided test set and validation set. Third, we found that on the basis of demographic characteristics, medical history, and mean arterial pressure, the addition of uric acid can improve the prediction ability of the model from the first trimester; the addition of platelet and alkaline phosphatase can improve the prediction ability of the model in the second and third trimester, respectively. We did not test the predictive value of uric acid in the second trimester, because pregnant women rarely did biochemical tests in this period. We suggest that pregnant women in the second trimester do additional uric acid tests in the future. In particular, at 28–31 weeks, 32–35 weeks and 36–39 weeks of pregnancy, the AUROC of the prediction model for all pre-eclampsia reached 0.86, 0.89 and 0.95, respectively, which has the value in predicting late-onset and term pre-eclampsia. Fourth, to our knowledge, our study is the first study to develop multi-stage prediction models and propose a screening scheme based on the Chinese women. The screening time covers almost the entire pregnancy period: the earliest to 5–10 weeks of pregnancy and the latest to 36–39 weeks of pregnancy.

Several clinical values can be seen in our study. First, we found that uric acid, platelets and alkaline phosphatase can improve the predictive ability of the model, which are part of the routine test items and it does not need extra costs. Second, a screening system has been developed for the Chinese population and policy environment, and the prediction window has been advanced to 5–10 weeks, which allows time for aspirin intervention and other means for high-risk groups. There were strong evidences supporting that aspirin is the only drug preventing pre-eclampsia (54). Some guidelines recommended women with high or moderate clinical risk factors to use aspirin starting at the first or second trimester (1, 35, 36, 55). ACOG recommended using aspirin starting between 12 and 28 weeks of gestation, ideally before 16 weeks, and ISSHP also supported it was ideally used before 16 weeks (1, 55). A clinical trial involving 14361 women suggested that using low-dose aspirin starting at 6–13 weeks of gestation could reduce risk of preterm pre-eclampsia and perinatal mortality in low-income and middle-income countries (56). The study advanced the recommended time of aspirin use to 6 weeks of gestation. Third, for women identified at high risks during the second or third trimester, and missing the best window for using aspirin, clinicians could take intensive surveillance or hospitalization, and carefully select the best delivery time for pregnant women. Some previous studies developed prediction models in the second or third trimester (43, 57–60). It is useful to develop multi-stage prediction models covering the first, second, and third trimester. Forth, in the third trimester of pregnancy, the prediction ability of late-onset and term pre-eclampsia was improved by adding uric acid and alkaline phosphatase. Although late-onset and term pre-eclampsia have a lower hazard than early-onset, preterm pre-eclampsia, the former two subtypes have a higher incidence rate. Doctors can treat pre-eclampsia by delivery, however, the early birth of newborns may still be detrimental to their future growth.

However, the study has several limitations. First, the study had a large sample size and a large number of outcomes, but the incidence rate of early-onset pre-eclampsia was small, because the incidence rate of early-onset pre-eclampsia was particularly low. We used LASSO for variable selection, so it was less likely to cause over fitting. Second, there were missing values in biomarkers, but it was in line with the actual clinical situation. We used a simple imputation method, that is, using the previous tested values to impute the missing values. This method is easy for health professionals to use. Third, we did not conduct external validation. But we conducted internal validation on the test set and validation set by randomly dividing the data set. Moreover, we used LASSO to select variables, which reduced the possibility of over fitting. In the future, our model awaits validation on other data sets.

## Data Availability Statement

The data analyzed in this study is subject to the following licenses/restrictions the data that support the findings of this study are available from Tongzhou Maternal and Child Health Care Hospital of Beijing but restrictions apply to the availability of these data, which were used under license for the current study, and so are not publicly available. Data are however available from the authors upon reasonable request and with permission of Tongzhou Maternal and Child Health Care Hospital of Beijing. Requests to access these datasets should be directed to Haijun Wang, whjun@pku.edu.cn.

## Ethics Statement

The studies involving human participants were reviewed and approved by Institutional Review Board of Peking University Health Science Center (No. IRB00001052-21023). Written informed consent for participation was not required for this study in accordance with the national legislation and the institutional requirements.

## Author Contributions

ZT, YJ, SZ, TS, ZY, and NH: data curation. ZT, YJ, JJ, and HW: methodology. ZT and JJ: software. ZT: writing—original draft. YJ, JJ, and HW: writing—review & editing. All authors contributed to the article and approved the submitted version.

## Funding

This study was funded by Beijing Natural Science Foundation (7212144) and National Natural Science Foundation of China (92046019).

## Conflict of Interest

The authors declare that the research was conducted in the absence of any commercial or financial relationships that could be construed as a potential conflict of interest.

## Publisher's Note

All claims expressed in this article are solely those of the authors and do not necessarily represent those of their affiliated organizations, or those of the publisher, the editors and the reviewers. Any product that may be evaluated in this article, or claim that may be made by its manufacturer, is not guaranteed or endorsed by the publisher.
