# Supplementary material for: Development and Validation of Multi-Stage Prediction Models for Pre-eclampsia: A Retrospective Cohort Study on Chinese Women
Source: Front Public Health. 2022 May 30;10:911975. doi: 10.3389/fpubh.2022.911975 (PMC9195617; doi:10.3389/fpubh.2022.911975)
Supplement: Supplementary file 1 [file Table_1.docx]

**Supplementary**

**Development and Validation of Multi-Stage Prediction Models for Pre-eclampsia: A retrospective cohort study on Chinese Women**

**TABLE1a Biomarkers from routine blood tests as candidate variables in developing model**

| **Item Number** | **Item Name** | **Unit** |
| --- | --- | --- |
| **1** | **Hematocrit** | **%** |
| **2** | **hemoglobin** | **g/L** |
| **3** | **Mean red blood cell volume** | **fL** |
| **4** | **platelet** | **10^9/L** |
| **5** | **red blood cell** | **10^12/L** |
| **6** | **white blood cell** | **10^9/L** |
| **7** | **Basophil ratio** | **%** |
| **8** | **Eosinophil ratio** | **%** |
| **9** | **Lymphocyte ratio** | **%** |
| **10** | **Mean hemoglobin** | **pg** |
| **11** | **Mean hemoglobin concentration** | **g/L** |
| **12** | **Monocyte ratio** | **%** |
| **13** | **Mean platelet volume** | **fL** |
| **14** | **Neutrophil ratio** | **%** |
| **15** | **Platelet hematocrit** | **%** |
| **16** | **Platelet distribution width** | **%** |
| **17** | **Large platelet ratio** | **%** |
| **18** | **Red blood cell distribution width** | **%** |

**TABLE1b Biomarkers from biochemical tests as candidate variables in developing model**

| **Item Number** | **Item Name** | **Unit** |
| --- | --- | --- |
| **1** | **alkaline phosphatase** | **U/L** |
| **2** | **Alanine aminotransferase** | **U/L** |
| **3** | **Aspartate aminotransferase** | **U/L** |
| **4** | **Urea nitrogen** | **mmol/L** |
| **5** | **calcium** | **mmol/L** |
| **6** | **total cholesterol** | **mmol/L** |
| **7** | **creatine kinase** | **U/L** |
| **8** | **chlorine** | **mmol/L** |
| **9** | **creatinine** | **μmol/L** |
| **10** | **glucose** | **mmol/L** |
| **11** | **potassium** | **mmol/L** |
| **12** | **lactate dehydrogenase** | **U/L** |
| **13** | **sodium** | **mmol/L** |
| **14** | **phosphorus** | **mmol/L** |
| **15** | **total bilirubin** | **μmol/L** |
| **16** | **triglyceride** | **mmol/L** |
| **17** | **Total protein** | **g/L** |
| **18** | **uric acid** | **μmol/L** |
| **19** | **Glutamyl transpeptidase** | **U/L** |
| **20** | **Albumin globulin ratio** | **——** |
| **21** | **amylase** | **U/L** |
| **22** | **creatine kinase isoenzyme** | **U/L** |
| **23** | **Carbon dioxide binding force** | **mmol/L** |
| **24** | **Direct bilirubin** | **μmol/L** |
| **25** | **Globulin** | **g/l** |
| **26** | **α- Hydroxybutyrate dehydrogenase** | **U/L** |
| **27** | **High density lipoprotein cholesterol** | **mmol/L** |
| **28** | **Indirect bilirubin** | **μmol/L** |
| **29** | **Low density lipoprotein cholesterol** | **mmol/L** |
| **30** | **Total bile acid** | **μmol/L** |

**Table 2a Performance of Prediction Model based on Characteristics and Medical History for All, Early-onset and Preterm Pre-eclampsia**

| **Gestational age  of prediction  (weeks)** | **All Pre-eclampsia** | | | | | **Early-Onset Pre-eclampsia** | | | | | **Preterm Pre-eclampsia** | | | | |
| --- | --- | --- | --- | --- | --- | --- | --- | --- | --- | --- | --- | --- | --- | --- | --- |
|  | **Variables** | **Sample Size** | **Cases(N)** | **Cases(%)** | **AUROC** | **Variables** | **Sample Size** | **Cases(N)** | **Cases(%)** | **AUROC** | **Variables** | **Sample Size** | **Cases(N)** | **Cases(%)** | **AUROC** |
| **<20** | **Basic Variables** | **15665** | **548** | **3.50%** | **0.68** | **Basic Variables** | **15665** | **39** | **0.25%** | **0.73** | **Basic Variables** | **15665** | **95** | **0.61%** | **0.74** |
| **<20** | **+ Height** | **15665** | **548** | **3.50%** | **0.68** | **+ Season** | **15665** | **39** | **0.25%** | **0.71** | **+ Season** | **15665** | **95** | **0.61%** | **0.72** |
| **<20** | **+ Height+ Season** | **15665** | **548** | **3.50%** | **0.68** | **+ Height+ Season** | **15665** | **39** | **0.25%** | **0.71** | **+ Height+ Season** | **15665** | **95** | **0.61%** | **0.72** |
| **24-27** | **Basic Variables** | **15644** | **527** | **3.37%** | **0.68** | **Basic Variables** | **15644** | **25** | **0.16%** | **0.69** | **Basic Variables** | **15644** | **81** | **0.52%** | **0.74** |
| **24-27** | **+ Height** | **15644** | **527** | **3.37%** | **0.68** | **+ Height** | **15644** | **25** | **0.16%** | **0.68** | **+ Height** | **15644** | **81** | **0.52%** | **0.72** |
| **24-27** | **+ Height+ Season** | **15644** | **527** | **3.37%** | **0.68** | **+ Height** | **15644** | **25** | **0.16%** | **0.68** | **+ Height** | **15644** | **81** | **0.52%** | **0.72** |

**Note: Basic Variables include pre-pregnancy BMI, pre-gestational diabetes mellitus, parity, family history of hypertension and maternal age, which are forced into models. Gestational diabetes mellitus as a candidate variable is not included in the final model at 24-27 weeks.**

**Table 2b Performance of Prediction Model based on Characteristics and Medical History for Late-onset and Term Pre-eclampsia**

| **Gestational age  of prediction  (weeks)** | **Late-Onset Pre-eclampsia** | | | | | **Term Pre-eclampsia** | | | | |
| --- | --- | --- | --- | --- | --- | --- | --- | --- | --- | --- |
|  | **Variables** | **Sample Size** | **Cases(N)** | **Cases(%)** | **AUROC** | **Variables** | **Sample Size** | **Cases(N)** | **Cases(%)** | **AUROC** |
| **<20** | **Basic Variables** | **15665** | **509** | **3.25%** | **0.68** | **Basic Variables** | **15665** | **453** | **2.89%** | **0.67** |
| **<20** | **+ Height** | **15665** | **509** | **3.25%** | **0.68** | **+ Height** | **15665** | **453** | **2.89%** | **0.67** |
| **<20** | **+ Height+ Season** | **15665** | **509** | **3.25%** | **0.67** | **+ Height+ Season** | **15665** | **453** | **2.89%** | **0.67** |
| **24-27** | **Basic Variables** | **15644** | **502** | **3.21%** | **0.68** | **Basic Variables** | **15644** | **446** | **2.85%** | **0.68** |
| **24-27** | **+ Height** | **15644** | **502** | **3.21%** | **0.68** | **+ Height** | **15644** | **446** | **2.85%** | **0.68** |
| **24-27** | **+ Height+ Season** | **15644** | **502** | **3.21%** | **0.68** | **+ Height+ Season** | **15644** | **446** | **2.85%** | **0.67** |

**Note: Basic Variables include pre-pregnancy BMI, pre-gestational diabetes mellitus, parity, family history of hypertension and maternal age, which are forced into models. Gestational diabetes mellitus as a candidate variable is not included in the final model at 24-27 weeks.**

**Table 3a Performance of Prediction Model based on Characteristics, Medical History, Mean Arterial Pressure for All, Early-onset and**

**Preterm Pre-eclampsia**

| **Gestational age of prediction (weeks)** | **Variables** | **All Pre-eclampsia** | | | | **Early-Onset Pre-eclampsia** | | | | **Preterm Pre-eclampsia** | | | |
| --- | --- | --- | --- | --- | --- | --- | --- | --- | --- | --- | --- | --- | --- |
|  |  | **Sample Size** | **Cases(N)** | **Cases(%)** | **AUROC** | **Sample Size** | **Cases(N)** | **Cases(%)** | **AUROC** | **Sample Size** | **Cases(N)** | **Cases(%)** | **AUROC** |
| **<20** | **Basic Variables** | **15665** | **548** | **3.50%** | **0.68** | **15665** | **39** | **0.25%** | **0.73** | **15665** | **95** | **0.61%** | **0.74** |
| **5-10** | **+MAP(5-10)** | **15665** | **548** | **3.50%** | **0.70** | **15665** | **39** | **0.25%** | **0.82** | **15665** | **95** | **0.61%** | **0.78** |
| **11-13** | **+MAPs(5-10,11-13)** | **15665** | **548** | **3.50%** | **0.74** | **15665** | **39** | **0.25%** | **0.78** | **15665** | **95** | **0.61%** | **0.77** |
| **14-18** | **+MAPs(5-10,14-18)** | **15665** | **548** | **3.50%** | **0.76** | **15665** | **39** | **0.25%** | **0.87** | **15665** | **95** | **0.61%** | **0.81** |
|  | **+MAPs(5-10,11-13,14-18)** | **15665** | **548** | **3.50%** | **0.76** | **15665** | **39** | **0.25%** | **0.86** | **15665** | **95** | **0.61%** | **0.81** |
| **19-23** | **+MAPs(5-10,19-23)** | **15655** | **538** | **3.44%** | **0.76** | **15655** | **32** | **0.20%** | **0.83** | **15655** | **88** | **0.56%** | **0.81** |
|  | **+MAPs(5-10,11-13,14-18,19-23)** | **15655** | **538** | **3.44%** | **0.77** | **15655** | **32** | **0.20%** | **0.86** | **15655** | **88** | **0.56%** | **0.81** |
| **24-27** | **+MAPs(5-10,24-27)** | **15644** | **527** | **3.37%** | **0.77** | **15644** | **25** | **0.16%** | **0.85** | **15644** | **81** | **0.52%** | **0.82** |
|  | **+MAPs(5-10,11-13,14-18,19-23,24-27)** | **15644** | **527** | **3.37%** | **0.79** | **15644** | **25** | **0.16%** | **0.85** | **15644** | **81** | **0.52%** | **0.82** |
| **28-31** | **+MAPs(5-10,28-31)** | **15619** | **504** | **3.23%** | **0.78** | **15619** | **10** | **0.06%** | **0.89** | **15619** | **65** | **0.42%** | **0.80** |
|  | **+MAPs(5-10,11-13,14-18,19-23,24-27,28-31)** | **15619** | **504** | **3.23%** | **0.80** | **15619** | **10** | **0.06%** | **0.88** | **15619** | **65** | **0.42%** | **0.80** |
| **32-35** | **+MAPs(5-10,32-35)** | **15478** | **459** | **2.97%** | **0.77** |  |  |  |  |  |  |  |  |
|  | **+MAPs(5-10,11-13,14-18,19-23,24-27,28-31,32-35)** | **15478** | **459** | **2.97%** | **0.78** |  |  |  |  |  |  |  |  |
| **36-39** | **+MAPs(5-10,36-39)** | **10912** | **131** | **1.20%** | **0.78** |  |  |  |  |  |  |  |  |
|  | **+MAPs(5-10,11-13,14-18,19-23,24-27,28-31,32-35,36-39)** | **10912** | **131** | **1.20%** | **0.76** |  |  |  |  |  |  |  |  |

**Note: Basic Variables include pre-pregnancy BMI, pre-gestational diabetes mellitus, parity, family history of hypertension and maternal age, which are forced into models. MAP: Mean Arterial Pressure. Numbers in brackets represent the gestational age when MAPS were measured.**

**Table 3b Performance of Prediction Model based on Characteristics, Medical History, Mean Arterial Pressure for Late-onset and**

**Term Pre-eclampsia**

| **Gestational age of prediction (weeks)** | **Variables** | **Late-Onset Pre-eclampsia** | | | | **Term Pre-eclampsia** | | | |
| --- | --- | --- | --- | --- | --- | --- | --- | --- | --- |
|  |  | **Sample Size** | **Cases(N)** | **Cases(%)** | **AUROC** | **Sample Size** | **Cases(N)** | **Cases(%)** | **AUROC** |
| **<20** | **Basic Variables** | **15665** | **509** | **3.25%** | **0.68** | **15665** | **453** | **2.89%** | **0.67** |
| **5-10** | **+MAP(5-10)** | **15665** | **509** | **3.25%** | **0.69** | **15665** | **453** | **2.89%** | **0.68** |
| **11-13** | **+MAPs(5-10,11-13)** | **15665** | **509** | **3.25%** | **0.73** | **15665** | **453** | **2.89%** | **0.73** |
| **14-18** | **+MAPs(5-10,14-18)** | **15665** | **509** | **3.25%** | **0.75** | **15665** | **453** | **2.89%** | **0.75** |
|  | **+MAPs(5-10,11-13,14-18)** | **15665** | **509** | **3.25%** | **0.76** | **15665** | **453** | **2.89%** | **0.75** |
| **19-23** | **+MAPs(5-10,19-23)** | **15655** | **506** | **3.23%** | **0.76** | **15655** | **450** | **2.87%** | **0.75** |
|  | **+MAPs(5-10,11-13,14-18,19-23)** | **15655** | **506** | **3.23%** | **0.77** | **15655** | **450** | **2.87%** | **0.76** |
| **24-27** | **+MAPs(5-10,24-27)** | **15644** | **502** | **3.21%** | **0.76** | **15644** | **446** | **2.85%** | **0.76** |
|  | **+MAPs(5-10,11-13,14-18,19-23,24-27)** | **15644** | **502** | **3.21%** | **0.78** | **15644** | **446** | **2.85%** | **0.78** |
| **28-31** | **+MAPs(5-10,28-31)** | **15619** | **494** | **3.16%** | **0.77** | **15619** | **439** | **2.81%** | **0.77** |
|  | **+MAPs(5-10,11-13,14-18,19-23,24-27,28-31)** | **15619** | **494** | **3.16%** | **0.79** | **15619** | **439** | **2.81%** | **0.79** |
| **32-35** | **+MAPs(5-10,32-35)** |  |  |  |  | **15478** | **429** | **2.77%** | **0.77** |
|  | **+MAPs(5-10,11-13,14-18,19-23,24-27,28-31,32-35)** |  |  |  |  | **15478** | **429** | **2.77%** | **0.78** |
| **36-39** | **+MAPs(5-10,36-39)** |  |  |  |  |  |  |  |  |
|  | **+MAPs(5-10,11-13,14-18,19-23,24-27,28-31,32-35,36-39)** |  |  |  |  |  |  |  |  |

**Note: Basic Variables include pre-pregnancy BMI, pre-gestational diabetes mellitus, parity, family history of hypertension and maternal age, which are forced into models. MAP: Mean Arterial Pressure. Numbers in brackets represent the gestational age when MAPS were measured.**

**Table 4a Performance of Prediction Model based on Characteristics, Medical History, Mean Arterial Pressure, and Biomarkers for All Pre-eclampsia in Women with and without Using Aspirin**

| Gestational age of prediction (weeks) | Variables | All Pre-eclampsia(With the Use of Aspirin) | | | | All Pre-eclampsia(Without the Use of Aspirin) | | | |
| --- | --- | --- | --- | --- | --- | --- | --- | --- | --- |
|  |  | Sample Size | Cases(N) | Cases(%) | AUROC | Sample Size | Cases(N) | Cases(%) | AUROC |
| <20 | Basic Variables | 15665 | 548 | 3.50% | 0.70 | 15561 | 530 | 3.41% | 0.69 |
| 5-10 | +MAP(5-10) | 15665 | 548 | 3.50% | 0.70 | 15561 | 530 | 3.41% | 0.69 |
|  | +MAP(5-10)+uric acid(5-10) | 15665 | 548 | 3.50% | 0.71 | 15561 | 530 | 3.41% | 0.71 |
| 11-13 | +MAPs(5-10,11-13) | 15665 | 548 | 3.50% | 0.74 | 15561 | 530 | 3.41% | 0.73 |
|  | +MAPs(5-10,11-13)+uric acid(11-13) | 15665 | 548 | 3.50% | 0.75 | 15561 | 530 | 3.41% | 0.74 |
| 14-18 | +MAPs(5-10,14-18) | 15665 | 548 | 3.50% | 0.76 | 15561 | 530 | 3.41% | 0.75 |
| 19-23 | +MAPs(5-10,19-23) | 15655 | 538 | 3.44% | 0.76 | 15551 | 520 | 3.34% | 0.76 |
|  | +MAPs(5-10,19-23)+platelets(5-10)+platelets(19-23) | 15655 | 538 | 3.44% | 0.79 | 15551 | 520 | 3.34% | 0.78 |
| 24-27 | +MAPs(5-10,24-27) | 15644 | 527 | 3.37% | 0.77 | 15542 | 511 | 3.29% | 0.76 |
|  | +MAPs(5-10,24-27)+platelets(5-10)+platelets(24-27) | 15644 | 527 | 3.37% | 0.80 | 15542 | 511 | 3.29% | 0.80 |
| 28-31 | +MAPs(5-10,28-31) | 15619 | 504 | 3.23% | 0.78 | 15520 | 491 | 3.16% | 0.77 |
|  | +MAPs(5-10,28-31)+uric acid(28-31) | 15619 | 504 | 3.23% | 0.79 | 15520 | 491 | 3.16% | 0.79 |
|  | +MAPs(5-10,28-31)+alkaline phosphatase(28-31) | 15619 | 504 | 3.23% | 0.85 | 15520 | 491 | 3.16% | 0.84 |
|  | +MAPs(5-10,28-31)+uric acid(28-31)+alkaline phosphatase(28-31) | 15619 | 504 | 3.23% | 0.86 | 15520 | 491 | 3.16% | 0.86 |
| 32-35 | +MAPs(5-10,32-35) | 15478 | 459 | 2.97% | 0.77 | 15384 | 451 | 2.93% | 0.77 |
|  | +MAPs(5-10,32-35)+uric acid(32-35) | 15478 | 459 | 2.97% | 0.80 | 15384 | 451 | 2.93% | 0.79 |
|  | +MAPs(5-10,32-35)+alkaline phosphatase(32-35) | 15478 | 459 | 2.97% | 0.87 | 15384 | 451 | 2.93% | 0.87 |
|  | +MAPs(5-10,32-35)+uric acid(32-35)+alkaline phosphatase(32-35) | 15478 | 459 | 2.97% | 0.89 | 15384 | 451 | 2.93% | 0.89 |
| 36-39 | +MAPs(5-10,36-39) | 10912 | 131 | 1.20% | 0.78 | 10863 | 130 | 1.20% | 0.78 |
|  | +MAPs(5-10,36-39)+uric acid(36-39) | 10912 | 131 | 1.20% | 0.82 | 10863 | 130 | 1.20% | 0.82 |
|  | +MAPs(5-10,36-39)+alkaline phosphatase(36-39) | 10912 | 131 | 1.20% | 0.93 | 10863 | 130 | 1.20% | 0.93 |
|  | +MAPs(5-10,36-39)+alkaline phosphatase(36-39)+uric acid(36-39) | 10912 | 131 | 1.20% | 0.95 | 10863 | 130 | 1.20% | 0.95 |

**Note: Basic Variables include pre-pregnancy BMI, pre-gestational diabetes mellitus, parity, family history of hypertension and maternal age, which are forced into models. MAP: Mean Arterial Pressure. Numbers in brackets represent the gestational age when MAPS and biomarkers were tested.**

**Table 4b Performance of Prediction Model based on Characteristics, Medical History, Mean Arterial Pressure, and Biomarkers for Early-onset Pre-eclampsia in Women with and without Using Aspirin**

| Gestational age of prediction (weeks) | Variables | Early-Onset Pre-eclampsia(With the Use of Aspirin) | | | | Early-Onset Pre-eclampsia(Without the Use of Aspirin) | | | |
| --- | --- | --- | --- | --- | --- | --- | --- | --- | --- |
|  |  | Sample Size | Cases(N) | Cases(%) | AUROC | Sample Size | Cases(N) | Cases(%) | AUROC |
| <20 | Basic Variables | 15665 | 39 | 0.25% | 0.82 | 15561 | 34 | 0.22% | 0.81 |
| 5-10 | +MAP(5-10) | 15665 | 39 | 0.25% | 0.82 | 15561 | 34 | 0.22% | 0.81 |
|  | +MAP(5-10)+uric acid(5-10) | 15665 | 39 | 0.25% | 0.82 | 15561 | 34 | 0.22% | 0.82 |
| 11-13 | +MAPs(5-10,11-13) | 15665 | 39 | 0.25% | 0.78 | 15561 | 34 | 0.22% | 0.76 |
|  | +MAPs(5-10,11-13)+uric acid(11-13) | 15665 | 39 | 0.25% | 0.78 | 15561 | 34 | 0.22% | 0.75 |
| 14-18 | +MAPs(5-10,14-18) | 15665 | 39 | 0.25% | 0.87 | 15561 | 34 | 0.22% | 0.85 |
| 19-23 | +MAPs(5-10,19-23) | 15655 | 32 | 0.20% | 0.83 | 15551 | 27 | 0.17% | 0.82 |
|  | +MAPs(5-10,19-23)+platelets(5-10)+platelets(19-23) | 15655 | 32 | 0.20% | 0.85 | 15551 | 27 | 0.17% | 0.83 |
| 24-27 | +MAPs(5-10,24-27) | 15644 | 25 | 0.16% | 0.85 | 15542 | 21 | 0.14% | 0.82 |
|  | +MAPs(5-10,24-27)+platelets(5-10)+platelets(24-27) | 15644 | 25 | 0.16% | 0.86 | 15542 | 21 | 0.14% | 0.83 |
| 28-31 | +MAPs(5-10,28-31) | 15619 | 10 | 0.06% | 0.89 | 15520 | 9 | 0.06% | 0.87 |
|  | +MAPs(5-10,28-31)+uric acid(28-31) | 15619 | 10 | 0.06% | 0.89 | 15520 | 9 | 0.06% | 0.88 |
|  | +MAPs(5-10,28-31)+alkaline phosphatase(28-31) | 15619 | 10 | 0.06% | 0.94 | 15520 | 9 | 0.06% | 0.93 |
|  | +MAPs(5-10,28-31)+uric acid(28-31)+alkaline phosphatase(28-31) | 15619 | 10 | 0.06% | 0.96 | 15520 | 9 | 0.06% | 0.95 |

**Note: Basic Variables include pre-pregnancy BMI, pre-gestational diabetes mellitus, parity, family history of hypertension and maternal age, which are forced into models. MAP: Mean Arterial Pressure. Numbers in brackets represent the gestational age when MAPS and biomarkers were tested.**

**Table 4c Performance of Prediction Model based on Characteristics, Medical History, Mean Arterial Pressure, and Biomarkers for Preterm Pre-eclampsia in Women with and without Using Aspirin**

| Gestational age of prediction (weeks) | Variables | Preterm Pre-eclampsia(With the Use of Aspirin) | | | | Preterm Pre-eclampsia(Without the Use of Aspirin) | | | |
| --- | --- | --- | --- | --- | --- | --- | --- | --- | --- |
|  |  | Sample Size | Cases(N) | Cases(%) | AUROC | Sample Size | Cases(N) | Cases(%) | AUROC |
| <20 | Basic Variables | 15665 | 95 | 0.61% | 0.78 | 15561 | 85 | 0.55% | 0.75 |
| 5-10 | +MAP(5-10) | 15665 | 95 | 0.61% | 0.78 | 15561 | 85 | 0.55% | 0.75 |
|  | +MAP(5-10)+uric acid(5-10) | 15665 | 95 | 0.61% | 0.80 | 15561 | 85 | 0.55% | 0.77 |
| 11-13 | +MAPs(5-10,11-13) | 15665 | 95 | 0.61% | 0.77 | 15561 | 85 | 0.55% | 0.74 |
|  | +MAPs(5-10,11-13)+uric acid(11-13) | 15665 | 95 | 0.61% | 0.78 | 15561 | 85 | 0.55% | 0.74 |
| 14-18 | +MAPs(5-10,14-18) | 15665 | 95 | 0.61% | 0.81 | 15561 | 85 | 0.55% | 0.77 |
| 19-23 | +MAPs(5-10,19-23) | 15655 | 88 | 0.56% | 0.81 | 15551 | 78 | 0.50% | 0.80 |
|  | +MAPs(5-10,19-23)+platelets(5-10)+platelets(19-23) | 15655 | 88 | 0.56% | 0.83 | 15551 | 78 | 0.50% | 0.82 |
| 24-27 | +MAPs(5-10,24-27) | 15644 | 81 | 0.52% | 0.82 | 15542 | 72 | 0.46% | 0.80 |
|  | +MAPs(5-10,24-27)+platelets(5-10)+platelets(24-27) | 15644 | 81 | 0.52% | 0.84 | 15542 | 72 | 0.46% | 0.82 |
| 28-31 | +MAPs(5-10,28-31) | 15619 | 65 | 0.42% | 0.80 | 15520 | 59 | 0.38% | 0.82 |
|  | +MAPs(5-10,28-31)+uric acid(28-31) | 15619 | 65 | 0.42% | 0.82 | 15520 | 59 | 0.38% | 0.81 |
|  | +MAPs(5-10,28-31)+alkaline phosphatase(28-31) | 15619 | 65 | 0.42% | 0.83 | 15520 | 59 | 0.38% | 0.83 |
|  | +MAPs(5-10,28-31)+uric acid(28-31)+alkaline phosphatase(28-31) | 15619 | 65 | 0.42% | 0.84 | 15520 | 59 | 0.38% | 0.82 |

**Note: Basic Variables include pre-pregnancy BMI, pre-gestational diabetes mellitus, parity, family history of hypertension and maternal age, which are forced into models. MAP: Mean Arterial Pressure. Numbers in brackets represent the gestational age when MAPS and biomarkers were tested.**

**Table 4d Performance of Prediction Model based on Characteristics, Medical History, Mean Arterial Pressure, and Biomarkers for Late-onset Pre-eclampsia in Women with and without Using Aspirin**

| Gestational age of prediction (weeks) | Variables | Late-Onset Pre-eclampsia(With the Use of Aspirin) | | | | Late-Onset Pre-eclampsia(Without the Use of Aspirin) | | | |
| --- | --- | --- | --- | --- | --- | --- | --- | --- | --- |
|  |  | Sample Size | Cases(N) | Cases(%) | AUROC | Sample Size | Cases(N) | Cases(%) | AUROC |
| <20 | Basic Variables | 15665 | 509 | 3.25% | 0.69 | 15561 | 496 | 3.19% | 0.68 |
| 5-10 | +MAP(5-10) | 15665 | 509 | 3.25% | 0.69 | 15561 | 496 | 3.19% | 0.68 |
|  | +MAP(5-10)+uric acid(5-10) | 15665 | 509 | 3.25% | 0.71 | 15561 | 496 | 3.19% | 0.70 |
| 11-13 | +MAPs(5-10,11-13) | 15665 | 509 | 3.25% | 0.73 | 15561 | 496 | 3.19% | 0.73 |
|  | +MAPs(5-10,11-13)+uric acid(11-13) | 15665 | 509 | 3.25% | 0.75 | 15561 | 496 | 3.19% | 0.74 |
| 14-18 | +MAPs(5-10,14-18) | 15665 | 509 | 3.25% | 0.75 | 15561 | 496 | 3.19% | 0.75 |
| 19-23 | +MAPs(5-10,19-23) | 15655 | 506 | 3.23% | 0.76 | 15551 | 493 | 3.17% | 0.75 |
|  | +MAPs(5-10,19-23)+platelets(5-10)+platelets(19-23) | 15655 | 506 | 3.23% | 0.79 | 15551 | 493 | 3.17% | 0.78 |
| 24-27 | +MAPs(5-10,24-27) | 15644 | 502 | 3.21% | 0.76 | 15542 | 490 | 3.15% | 0.76 |
|  | +MAPs(5-10,24-27)+platelets(5-10)+platelets(24-27) | 15644 | 502 | 3.21% | 0.80 | 15542 | 490 | 3.15% | 0.79 |
| 28-31 | +MAPs(5-10,28-31) | 15619 | 494 | 3.16% | 0.77 | 15520 | 482 | 3.11% | 0.77 |
|  | +MAPs(5-10,28-31)+uric acid(28-31) | 15619 | 494 | 3.16% | 0.79 | 15520 | 482 | 3.11% | 0.79 |
|  | +MAPs(5-10,28-31)+alkaline phosphatase(28-31) | 15619 | 494 | 3.16% | 0.84 | 15520 | 482 | 3.11% | 0.84 |
|  | +MAPs(5-10,28-31)+uric acid(28-31)+alkaline phosphatase(28-31) | 15619 | 494 | 3.16% | 0.86 | 15520 | 482 | 3.11% | 0.86 |

**Note: Basic Variables include pre-pregnancy BMI, pre-gestational diabetes mellitus, parity, family history of hypertension and maternal age, which are forced into models. MAP: Mean Arterial Pressure. Numbers in brackets represent the gestational age when MAPS and biomarkers were tested.**

**Table 4e Performance of Prediction Model based on Characteristics, Medical History, Mean Arterial Pressure, and Biomarkers for Term Pre-eclampsia in Women with and without Using Aspirin**

| Gestational age of prediction (weeks) | Variables | Term Pre-eclampsia(With the Use of Aspirin) | | | | Term Pre-eclampsia(Without the Use of Aspirin) | | | |
| --- | --- | --- | --- | --- | --- | --- | --- | --- | --- |
|  |  | Sample Size | Cases(N) | Cases(%) | AUROC | Sample Size | Cases(N) | Cases(%) | AUROC |
| <20 | Basic Variables | 15665 | 453 | 2.89% | 0.68 | 15561 | 445 | 2.86% | 0.68 |
| 5-10 | +MAP(5-10) | 15665 | 453 | 2.89% | 0.68 | 15561 | 445 | 2.86% | 0.68 |
|  | +MAP(5-10)+uric acid(5-10) | 15665 | 453 | 2.89% | 0.69 | 15561 | 445 | 2.86% | 0.70 |
| 11-13 | +MAPs(5-10,11-13) | 15665 | 453 | 2.89% | 0.73 | 15561 | 445 | 2.86% | 0.73 |
|  | +MAPs(5-10,11-13)+uric acid(11-13) | 15665 | 453 | 2.89% | 0.74 | 15561 | 445 | 2.86% | 0.74 |
| 14-18 | +MAPs(5-10,14-18) | 15665 | 453 | 2.89% | 0.75 | 15561 | 445 | 2.86% | 0.75 |
| 19-23 | +MAPs(5-10,19-23) | 15655 | 450 | 2.87% | 0.75 | 15551 | 442 | 2.84% | 0.75 |
|  | +MAPs(5-10,19-23)+platelets(5-10)+platelets(19-23) | 15655 | 450 | 2.87% | 0.78 | 15551 | 442 | 2.84% | 0.78 |
| 24-27 | +MAPs(5-10,24-27) | 15644 | 446 | 2.85% | 0.76 | 15542 | 439 | 2.82% | 0.76 |
|  | +MAPs(5-10,24-27)+platelets(5-10)+platelets(24-27) | 15644 | 446 | 2.85% | 0.79 | 15542 | 439 | 2.82% | 0.79 |
| 28-31 | +MAPs(5-10,28-31) | 15619 | 439 | 2.81% | 0.77 | 15520 | 432 | 2.78% | 0.77 |
|  | +MAPs(5-10,28-31)+uric acid(28-31) | 15619 | 439 | 2.81% | 0.78 | 15520 | 432 | 2.78% | 0.78 |
|  | +MAPs(5-10,28-31)+alkaline phosphatase(28-31) | 15619 | 439 | 2.81% | 0.84 | 15520 | 432 | 2.78% | 0.84 |
|  | +MAPs(5-10,28-31)+uric acid(28-31)+alkaline phosphatase(28-31) | 15619 | 439 | 2.81% | 0.86 | 15520 | 432 | 2.78% | 0.86 |
| 32-35 | +MAPs(5-10,32-35) | 15478 | 429 | 2.77% | 0.77 | 15384 | 424 | 2.76% | 0.77 |
|  | +MAPs(5-10,32-35)+uric acid(32-35) | 15478 | 429 | 2.77% | 0.80 | 15384 | 424 | 2.76% | 0.79 |
|  | +MAPs(5-10,32-35)+alkaline phosphatase(32-35) | 15478 | 429 | 2.77% | 0.87 | 15384 | 424 | 2.76% | 0.87 |
|  | +MAPs(5-10,32-35)+uric acid(32-35)+alkaline phosphatase(32-35) | 15478 | 429 | 2.77% | 0.89 | 15384 | 424 | 2.76% | 0.89 |

**Note: Basic Variables include pre-pregnancy BMI, pre-gestational diabetes mellitus, parity, family history of hypertension and maternal age, which are forced into models. MAP: Mean Arterial Pressure. Numbers in brackets represent the gestational age when MAPS and biomarkers were tested.**
